# Socioeconomic Inequalities in Alcohol and Tobacco Consumption: A National Ecological Study in Mexican Adolescents

**DOI:** 10.1155/2023/3604004

**Published:** 2023-07-03

**Authors:** Leticia Ávila-Burgos, Rebeca Guzmán-Saldaña, María de Lourdes Márquez-Corona, América Patricia Pontigo-Loyola, Sonia Márquez-Rodríguez, Mariana Mora-Acosta, Gladys Remigia Acuña-González, Antonio Hernández-Morales, Carlo Eduardo Medina-Solís

**Affiliations:** ^1^Health Systems Research Center, The National Institute of Public Health, Cuernavaca, Mexico; ^2^Academic Area of Psychology, Health Sciences Institute, Autonomous University of Hidalgo State, Pachuca, Mexico; ^3^Academic Area of Dentistry, Health Sciences Institute, Autonomous University of Hidalgo State, Pachuca, Mexico; ^4^School of Dentistry, Autonomous University of Campeche, Campeche, Mexico; ^5^School of Dentistry, Autonomous University of the State of Mexico, Toluca, Mexico; ^6^Advanced Studies and Research Center in Dentistry “Keisaburo Miyata”, School of Dentistry, Autonomous University of State of Mexico, Toluca, Mexico

## Abstract

Alcohol and cigarettes are the psychoactive substances that adolescents use most frequently. When both addictions are combined, they carry the worst burden of disease globally. The objective of this study was to identify whether socioeconomic factors correlate with alcohol and tobacco consumption in Mexican adolescents aged 10 years or more and to establish the relationship in the consumption between the two substances. This ecological study utilized data describing alcohol and tobacco consumption among adolescents aged 10–16 years (*n* = 48,837 ≈ *N* = 11,621,100). Having ever consumed any alcohol-containing beverage constituted alcohol consumption. Smoking a cigarette within 30 days constituted cigarette consumption. For both variables, the state-level percentages reported in the survey were used. Diverse socioeconomic variables were collected from official sources. Data on the prevalence of tobacco use and alcohol consumption were entered into an Excel database estimated for each of the states of the Mexican Republic, as well as the socioeconomic variables. We performed the analysis using Stata 14. Consumption prevalence was 15.0% for alcohol and 4.2% for tobacco. Alcohol consumption was not correlated with any studied socioeconomic variable (*p* > 0.05). The prevalence of tobacco consumption among elementary school students correlated (*p* < 0.05) with the portion of the population living in private dwellings without sewage, drainage, or sanitation (*r* = 0.3853). The prevalence of tobacco consumption among middle-school adolescents correlated with the portion of the employed population that earned up to two minimum wages (*r* = 0.3960), the percentage in poverty by income 2008 (*r* = 0.4754) and 2010 (*r* = 0.4531), and the percentage in extreme poverty by income 2008 (*r* = 0.4612) and 2010 (*r* = 0.4291). Positive correlations were found between tobacco consumption and alcohol consumption among both elementary (*r* = 0.5762, *p*=0.0006) and middle-school children (*r* = 0.7016, *p*=0.0000). These results suggest that certain socioeconomic factors correlate with tobacco consumption but not alcohol consumption. A correlation between alcohol consumption and tobacco consumption was observed. The results can be used for developing interventions in adolescents.

## 1. Introduction

Globally, just over half of the adolescents grow up in countries with a variety of health problems, including diseases of poverty, noncommunicable diseases (NCDs), and injuries. Similarly, unhealthy behaviors and exposure to various risk factors jeopardize not only their current health but also that of their adulthood, so morbidity and premature mortality remain considerable. In this regard, evidence has shown that adolescent health and development have an impact on the economic productivity, well-being, and development of the next generation [1–3].

Harmful alcohol consumption, understood as the intake that causes detrimental consequences on the drinker's health and environment [4, 5], is a serious public health problem associated with an increased risk of chronic diseases, injuries, and death. In fact, it is a causal factor in more than 200 diseases and disorders. In countries where NCDs and injuries are prevalent, rates of heavy drinking among adolescents were higher, with one-quarter of men and adolescents aged 15–25 years reporting drinking on at least one occasion in the past year [2]. This translates into a heavy social and economic burden for the world [6]. Alcohol consumption among adolescents is of increasing concern, as it reduces self-control and increases risky behaviors [7, 8]. Therefore, it contributes to high health care costs and loss of productivity and increases the likelihood of criminal activity and public disorder [1, 2, 9]. Contrary to what is observed in several European [10–14] and other developed countries [10, 15], in Mexico, various epidemiological sources allow us to take into account the growth that this pattern of consumption has had [16]. According to the National Survey on Drug, Alcohol and Tobacco Consumption [17], 71% of the population has consumed alcohol at some time in their lives. Of those who consumed alcohol, 53.1% reported having done so for the first time at 17 years of age or earlier. In adolescents, according to the National Health and Nutrition Survey 2018 (ENSANUT), the percentage of the population aged 10–19 years and older that consumes alcohol (last 30 days) was 21.7% [18] and the global prevalence of annual consumption was 38.3% [19].

According to the WHO, smoking represents an unprecedented public health threat [20]. It is associated with six of the eight leading causes of death in the world. The vast majority of people who use tobacco today started when they were adolescents. Globally, at least one in ten adolescents uses tobacco, and in some regions, this number is higher [20, 21]. Approximately 80% of smokers are from low- and middle-income countries, and the frequency of smoking among adolescents in these countries is high [21], representing the existence of socioeconomic inequalities. In Mexico, among the population aged 12–65 years, 40.9% and 35.6% reported having tried cigarettes at some time in their lives in 2002 and 2008, respectively. In 2008, 14.9% of adolescents reported having tried cigarettes at some time in their lives [22]. In 2018, the global prevalence of current tobacco use among adolescents was 5.7%, higher in males (8.8%) than in adolescent females (2.6%) [19].

The most commonly consumed psychoactive substances are tobacco and alcohol. These have the greatest weight in the global burden of disease when both addictions are added together. Individually, tobacco and alcohol use are the first and fourth leading causes of global loss of disability-adjusted life years, respectively [23]. Smoking and heavy alcohol use contribute significantly to health inequalities and cause significant harm to people other than their users. Children whose parents smoke are twice as likely to become smokers, and children whose parents consume alcohol are also more likely to drink in adolescence [9, 23]. For McKee and Weinberger [24], the relationship between tobacco and alcohol use has been little studied; they share a high degree of comorbidity and interact at many levels of analysis in the general population. Epidemiological data have shown that daily, nondaily, and current smokers are more likely to consume alcoholic beverages at different levels. There is now evidence that smoking increases the risk of alcohol abuse [24, 25], although reciprocal associations have also been proposed [26]. The relationship between smoking and alcoholism has been researched and exploited to such an extent that tobacco companies have even developed marketing strategies targeting both drinkers and drinking venues [24, 25].

The health-disease state of the population is the result of various variables and social determinants that originate in the structure and functioning of society. Social determinants of health are understood as all those circumstances in which people are born, grow, live, work, and age, as well as the systems established to combat diseases [27]. These circumstances largely determine the behaviors that people adopt and the decisions they make, shaped by a broader set of forces: economic, social, educational, regulatory, and political, among others [27–29]. Around the world, evidence has accumulated on the association between social determinants and a variety of health-related conditions. Health is distributed along a gradient in which people with lower socioeconomic status are more likely to suffer a higher burden of disease and earlier mortality than those with higher socioeconomic status [30–32]. The burden of disease caused by tobacco and alcohol use has a disproportionate impact on the poorest and least educated members of society relative to their more advantaged counterparts, a fact that is called “social gradient in health” [33–37].

Studies that analyze the problem of alcohol and tobacco consumption by adolescents are considered a priority in terms of public health [35, 38, 39] in order to generate data that can be used to promote policies that encourage the promotion and education of healthy lifestyle habits in this age group. Although there are several studies in Mexico on alcohol [40, 41] and tobacco [42, 43] consumption in Mexican adolescents, the relationship between these two events has been little explored [44]. The hypothesis of our analysis is that there are socioeconomic inequalities in the consumption of these substances and there is a close relationship between the estimated prevalences of tobacco consumption and alcohol consumption at the ecological level. The aim of the present study was to identify the existence of socioeconomic inequalities in the consumption of alcohol and tobacco in Mexican elementary and middle-school adolescents aged 10 years and older and to establish the relationship in the consumption between the two substances.

## 2. Materials and Methods

### 2.1. Study Design

Research with ecological design was carried out on a nationwide scale. The data were related to students who participated in the 2008 National School Health Survey (ENSE) between the ages of 10 and 16 [45]. Several results with mentions of the methodology have already been published [46, 47].

### 2.2. Description of the Origin Survey

The original data were obtained using the self-administration technique, that is, each student answered directly on a computer to protect privacy and confidentiality. The 32 states that make up the Mexican Republic participated in the ENSE, which is a nationally representative survey. Information was gathered from 73,560 children, representing 19,745,366 primary and middle-school students, at a total of 644 public schools. Interviews were conducted with 30,244 middle-school pupils and 43,316 elementary school students overall. The students who were included ranged in age from 5 to 16. Data on alcohol and tobacco use were collected only for those aged 10 years and older (*n* = 48,837 ≈ *N* = 11,621,100) [45].  Table 1 shows the distribution of adolescents.

### 2.3. Study Variables

Cases of tobacco use were defined on the basis of the number of cigarettes smoked in the last 30 days. The categories used were “smoker,” “nonsmoker,” and “never smoker.” A smoker is considered to be an adolescent who reported having smoked cigarettes during the 30 days prior to the survey, nonsmokers are those adolescents who reported not having smoked in the month prior to the survey, and never smokers are those who reported never having tried cigarettes [45]. Alcohol consumption, on the other hand, was considered when the adolescent answered that he or she had consumed a drink containing alcohol in his or her life [45]. For both variables, the state-level percentages reported in the survey were used.

As in other reports [46, 47], the socioeconomic variables on marginalization were obtained from the National Population Council (CONAPO) [48]: percentage of population aged 15 years or older illiterate, percentage of population aged 15 years or older without completed elementary school, percentage of occupants in inhabited private dwellings without piped water, percentage of occupants in inhabited private dwellings without sewage drainage or sanitation, percentage of occupants in inhabited private dwellings without electricity, percentage of occupants in inhabited private dwellings with dirt floors, percentage of occupants in inhabited private dwellings with some level of overcrowding, percentage of population in localities with less than five thousand inhabitants, percentage of employed population with an income of up to two minimum wages, and the state marginalization index [48].

In addition, two poverty indicators were used: the extreme poverty line by income, which is equivalent to the value of the food basket per person per month, and the poverty line by income, which is equivalent to the total value of the food basket and the nonfood basket per person per month. The percentage of poverty, extreme poverty, and the Gini index (measure of income concentration: it takes values between zero and one; when the value is close to one, it indicates that there is a higher income concentration. The Gini coefficient is a number between 0 and 1, where 0 corresponds to perfect equality (everyone has the same income) and 1 corresponds to perfect inequality (one person has all the income and the rest have none) were obtained from the National Council for the Evaluation of Social Development Policy (CONEVAL) [49].

### 2.4. Statistical Analysis

As the first step, the data on the prevalence of tobacco use and alcohol consumption were entered into an Excel database estimated for each of the states of the Mexican Republic, as well as the socioeconomic variables. Subsequently, statistical analysis was performed using Spearman's correlation test. Our analytical strategies were guided by results from bivariate analyses. A multivariate analysis could not be undertaken because the high correlation between socioeconomic variables was included. We performed the analysis using Stata 14.

### 2.5. Ethical Considerations

This study (ecological) was based on secondary data obtained from several publicly available datasets; therefore, direct informed consent was not required for this analysis, as established by the relevant institutional review board at the academic organization in which the lead author is employed (UAEH-ICSA-ODO-058). However, informed consent was obtained in the original study.

## 3. Results

Of the respondents, 47.1% were men and 52.9% were women. The results on alcohol and tobacco use in adolescents aged 10 years and older were collected from 48,837 individuals, representing approximately 11,621,100 adolescents nationwide.  Table 1 shows that, among elementary school students, slightly more than 70% are between 10 and 11 years old, while 78% of middle-school students are between 13 and 15 years old.

The prevalence of adolescents who reported having smoked cigarettes in the last month prior to the National Survey was 4.2%. This was higher in middle-school students (6.7%, CI 95% = 6.0–7.6) compared with elementary school students (1.8%, CI 95% = 1.5–2.3).  Table 2 shows the distribution of tobacco use across the 32 states that make up the Mexican Republic. In elementary school, the states with the lowest prevalence of tobacco use were Chiapas (0.0%), Sinaloa (0.3%), and Michoacán (0.4%), while the states with the highest tobacco use were Yucatán (4.6%), Hidalgo (5.4%), and Zacatecas (5.9%). In middle-school adolescents, the states with the lowest prevalence of tobacco use were Sinaloa (1.4%), Baja California Sur (1.7%), and Tamaulipas (2.8%), while Puebla (10.8%), Zacatecas (11.7%), and San Luis Potosí (11.9%) were the states with the highest tobacco use.

The estimated national prevalence of alcohol consumption at the time of the survey among students aged 10 years or older was 15.0%. The proportion of students with this risk behavior was higher among those attending middle school (24.0%, CI 95% = 22.0–26.0) compared with elementary school students (6.7%, CI 95% = 5.7–7.8).  Table 2 presents the distribution of alcohol consumption across the 32 states of the Mexican Republic. In elementary school, the states with the lowest prevalence of alcohol consumption were Chiapas (1.2%), Sinaloa (1.8%), and Tamaulipas (2.5%), while the states with the highest alcohol consumption were Guerrero (13.4%), Chihuahua (13.4%), and Zacatecas (13.8%). In middle school, the states with the lowest prevalence of tobacco use were Sinaloa (10.8%), Guanajuato (14.7%), and Tamaulipas (14.9%), while Mexico City (35.6%), Morelos (36.8%), and Zacatecas (43.9%) were the states with the highest alcohol consumption.

Table 3 presents the results of the correlations between tobacco and alcohol consumption in Mexican adolescents and the different socioeconomic variables. Alcohol consumption showed no correlation with any variable. On the other hand, tobacco use in elementary school students correlated with the percentage of occupants in inhabited private dwellings without sewage drainage or sanitation (*r* = 0.3853, *p*=0.0294). On the other hand, tobacco use in middle-school adolescents correlated with the percentage of the employed population with an income of up to two minimum wages (*r* = 0.3960, *p*=0.0249), the percentage of poverty by income 2008 (*r* = 0.4754, *p*=0.0060), the percentage of poverty by income 2010 (*r* = 0.4531, *p*=0.0092), the percentage of extreme poverty by income 2008 (*r* = 0.4612, *p*=0.0079), and the percentage of extreme poverty by income 2010 (*r* = 0.4291, *p*=0.0143).

**Figure 1:**
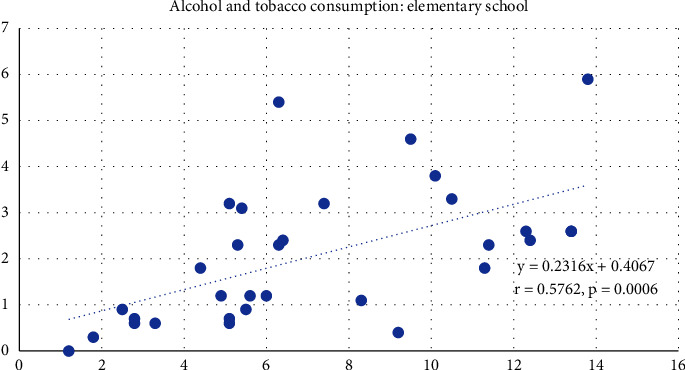
Correlation between tobacco and alcohol consumption among elementary students in Mexico.

**Figure 2:**
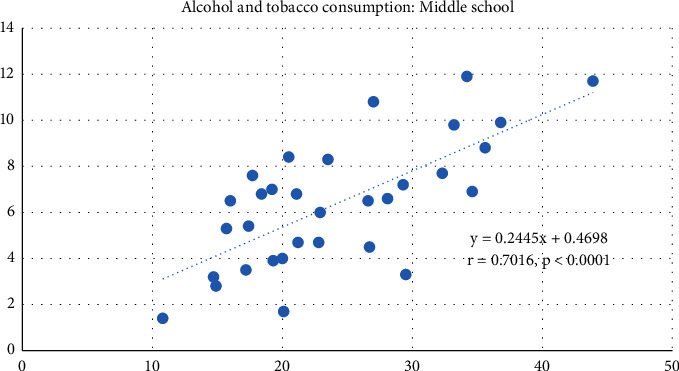
Correlation between tobacco and alcohol consumption among middle-school adolescents in Mexico.

Figures 1 and 2 show the results of the correlation analysis between tobacco and alcohol consumption in elementary (Figure 1) and middle-school (Figure 2) students, which shows a positive correlation, as the prevalence of tobacco consumption increases, so does the prevalence of alcohol consumption in both elementary (*r* = 0.5762, *p*=0.0006) and middle schools (*r* = 0.7016, *p* < 0.0001).

## 4. Discussion

This study aimed to identify the existence of socioeconomic inequalities in alcohol and tobacco consumption and of correlation at the ecological level of state prevalences of alcohol and tobacco consumption in Mexican elementary and middle-school adolescents aged 10–16 years, verifying the hypothesis that there is a positive and significant correlation between these two events. Although the results did not show the existence of socioeconomic inequalities in alcohol consumption, as observed in other studies, including prorich inequality [50–52], they were observed for tobacco consumption. We must take into account that there are a variety of factors at the individual/household level (for example, age, education, and income) and the societal level (for example, taxation policy, ease of alcohol availability, and norms around alcohol consumption) that influence alcohol/tobacco consumption. However, in the world, socioeconomic status remains one of the most important social determinants of alcohol-attributable harm [51]. Over time, certain variations in the prevalence of consumption of these substances have been reported in Mexico [17, 53]. The results demonstrate the existence of different nuances of tobacco and alcohol consumption in adolescents among the different states that make up the Mexican Republic, which had been little analyzed. Due to the fact that both behaviors are associated with a large number of conditions such as injuries, violence, and NCDs, their consumption among the adolescent population is of concern and makes them serious public health problems [54–56]. Although it has been reported that tobacco and alcohol consumption patterns in adolescents are qualitatively different from those of other age groups [44], it is also evident and worrying that adolescents are copying the drinking patterns of adults and that a significant proportion already have problems in their consumption patterns [22].

Alcohol consumption is one of the main risk factors for death and disability [6]. According to a report published by the WHO, in 2016, more than 3 million people died as a result of harmful alcohol consumption [57]. Thus, small reductions in health harms associated with low levels of alcohol intake are outweighed by the increased risk of other health-related harms. Indeed, a report published by the UK Medical Director found that “there is no safe level of alcohol consumption” and suggested that policies should prioritize reducing alcohol consumption at the population level [58]. For its part, an estimated 100 million people died from tobacco during the 20th century [21]; in 2017, about 8 million people died of a tobacco-related disease, and the WHO estimates point to nearly 10 million deaths in 2020 [20]. Early onset of alcohol and tobacco use is a risk factor; particularly in the case of alcohol [59], for heavy drinking in adulthood, they are also associated with an increased likelihood of using other drugs. The implications of the results have to do with the “Gateway Drug Theory,” which suggests that licit drugs, such as tobacco and alcohol, serve as a “gateway” to the use of other illicit drugs. In other words, the use of licit drugs (for example, alcohol and tobacco) will lead to the use of illicit substances (for example, cannabis, cocaine, or heroin) [56, 60–64]. However, there remains some disagreement as to which drug, alcohol, tobacco, or even marijuana, serves as the initial “gateway” drug that subsequently leads to the use of illicit drugs such as cocaine and heroin [65]. Given the current evidence, authors such as Bonevski et al. [66] point out that knowledge of the interactions between alcohol and tobacco can be used to reduce their use.

The results of the correlations show that tobacco consumption in Mexican adolescents presents certain health inequalities, since as the percentages of the worst conditions of the socioeconomic variables increased, so did the percentages of tobacco consumption. In this regard, previous studies have shown that tobacco consumption is disproportionately higher among lower socioeconomic groups [67–69]. This is consistent with the results observed in this study at the ecological level. Similarly, it has been observed that people with higher socioeconomic status are more likely to quit smoking among the general adult population [70]. In fact, of the millions of people that tobacco will kill in 2030, more than 80% will live in low- and middle-income countries. Therefore, in order to reduce the global burden of disease and death, effective tobacco control policies and programs need to be implemented [71]. To reduce the harmful effects of tobacco and alcohol consumption on the present and future health of adolescents, recommendations from around the world point to the importance of enforcing regulations that limit underage access to tobacco and alcohol through restrictions on the age of purchase, restricted hours of sale, and reducing demand by applying taxes that increase their prices [2, 22]. Positive correlations were found between tobacco consumption and alcohol consumption among both elementary and middle-school children. Some studies have observed an association between these two substances; the problem is that this situation increases the probability of co-use of other substances [60, 61].

When evaluating the findings of the current study, its limitations must be taken into consideration. First, because it is an ecological study, its design highlights the “ecological fallacy” problem, which is the false inference produced when wrong conclusions are drawn at the individual level (that is, about interindividual variability) from information at the group level [72]. It was also impossible to investigate the variations and connections between alcohol and cigarette use when sex was taken into account. On the other hand, given that pupils outside the 10–16 age group, in addition to not including those outside the national educational system, were not taken into consideration, there is the possibility that the data are endogeneous. Finally, since causality cannot be inferred from the analysis's contributing elements, the present findings should only be seen as statistical relationships. The findings, however, can be used to establish health policies or programs that attempt to reduce the use of either of the two chemicals under study. This has implications for public health policies and clinical treatment for tobacco and alcohol use, suggesting a greater emphasis on addressing multiple social and health concerns [65].

## 5. Conclusion

In conclusion, we can say that we observed a correlation between tobacco and alcohol consumption at the ecological level among Mexican adolescent school students aged 10 years and older. The results of the study underscore the importance of developing programs and interventions that do not target the use of a specific substance but rather address the use of multiple substances at the same time in the adolescent population.

## Figures and Tables

**Table 1:** Distribution of included adolescents by age group, Mexico.

Age group	Elementary school			Middle school		
	*n*	*N* (thousands)	%	*n*	*N* (thousands)	%
10 years	6,655	2,113.1	35.3	6	0.4	0.01
11 years	6,734	2,105.0	35.2	65	14.9	0.3
12 years	4,089	1,384.3	23.1	5,103	1,007.0	17.9
13 years	850	273.2	4.6	9,592	1,756.0	31.1
14 years	206	78.5	1.3	9,560	1,807.1	32.0
15 years	48	24.1	0.4	4,932	861.5	15.3
16 years	11	3.2	0.1	986	192.8	3.4
	18,593	5,981.4	100.0	30,244	5,639.7	100.0

**Table 2:** Prevalences of alcohol consumption (according to consumption of alcoholic beverages at some time in life) and tobacco use (according to cigarette consumption during the 30 days prior to the survey) in Mexican adolescents aged 10 years and older.

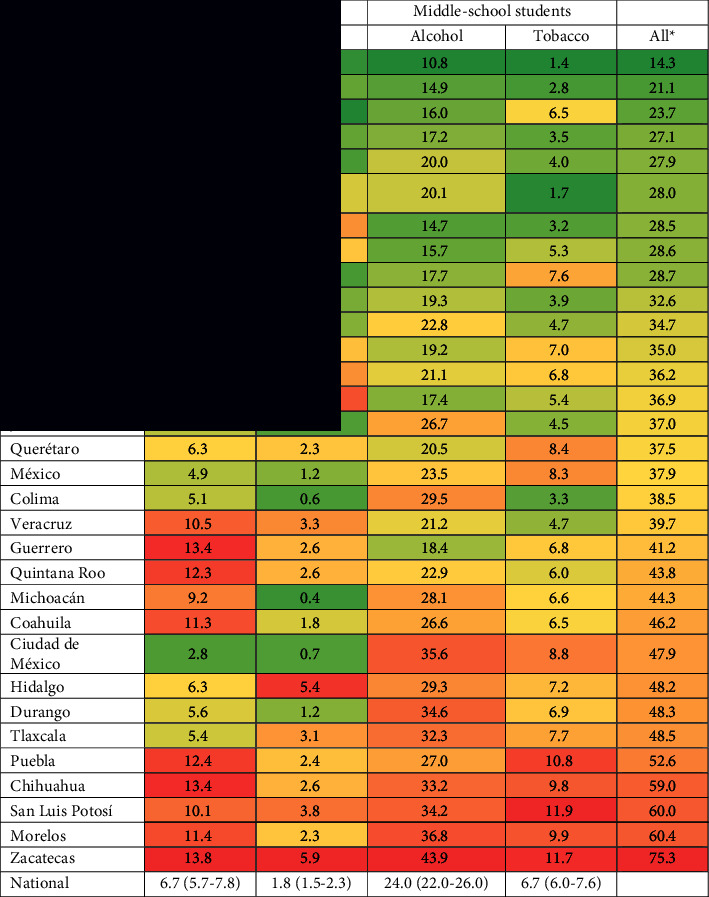

^*∗*^Combined prevalences of tobacco and alcohol consumption in elementary and middle-school adolescents in Mexico. The green tones indicate the lowest prevalences, while the red tones refer to the highest prevalences.

**Table 3:** Correlation of tobacco and alcohol consumption with the socioeconomic variables that make up the marginalization index, in addition to the Gini index, the percentage of poverty and extreme poverty.

		Elementary students		Middle-school students	
		Alcohol	Tobacco	Alcohol	Tobacco
V1	Spearman *r* =	0.1954	0.2534	−0.0939	0.2690
	*p* value =	0.2838	0.1616	0.6094	0.1366

V2	Spearman *r* =	0.1873	0.1585	−0.1136	0.1639
	*p* value =	0.3048	0.3863	0.5357	0.3701

V3	Spearman *r* =	0.2456	0.3853	−0.0645	0.2983
	*p* value =	0.1755	**0.0294**	0.7257	0.0973

V4	Spearman *r* =	0.1254	0.1921	−0.0770	0.1667
	*p* value =	0.4939	0.2922	0.6754	0.3620

V5	Spearman *r* =	0.1663	0.1803	−0.0026	0.3408
	*p* value =	0.3629	0.3233	0.9889	0.0563

V6	Spearman *r* =	0.0209	0.1796	−0.3383	0.1041
	*p* value =	0.9096	0.3253	0.0582	0.5706

V7	Spearman *r* =	0.0858	−0.0373	−0.0191	0.1458
	*p* value =	0.6404	0.8395	0.9175	0.4261

V8	Spearman *r* =	0.1293	0.1930	−0.0396	0.3313
	*p* value =	0.4806	0.2899	0.8297	0.0640

V9	Spearman *r* =	0.2126	0.3221	0.0297	0.3960
	*p* value =	0.2428	0.0722	0.8718	**0.0249**

V10	Spearman *r* =	0.2188	0.2938	−0.0678	0.3186
	*p* value =	0.2289	0.1026	0.7123	0.0755

V11	Spearman *r* =	0.2208	0.2405	0.0977	0.4754
	*p* value =	0.2245	0.1849	0.5948	**0.0060**

V12	Spearman *r* =	0.2025	0.2849	0.0643	0.4531
	*p* value =	0.2663	0.1140	0.7264	**0.0092**

V13	Spearman *r* =	0.2933	0.3267	0.0292	0.4612
	*p* value =	0.1032	0.0680	0.8742	**0.0079**

V14	Spearman *r* =	0.2220	0.3298	−0.0086	0.4291
	*p* value =	0.2219	0.0653	0.9627	**0.0143**

V15	Spearman *r* =	0.0147	−0.0185	−0.0858	0.1910
	*p* value =	0.9365	0.9197	0.6407	0.2949

V1 = percentage of population aged 15 years or older illiterate, V2 = percentage of population aged 15 years or older without completed elementary education, V3 = percentage of occupants in inhabited private dwellings without sewage drainage or sanitation, V4 = percentage of occupants in inhabited private dwellings without electricity, V5 = percentage of occupants in inhabited private dwellings without piped water, V6 = percentage of occupants in inhabited private dwellings with some level of overcrowding, V7 = percentage of occupants in inhabited private dwellings with dirt floor, V8 = percentage of population in localities with less than five thousand inhabitants, V9 = percentage of employed population with an income of up to two minimum wages, V10 = state marginalization index, V11 = poverty by income 2008, V12 = poverty by income 2010, V13 = extreme poverty by income 2008, V14 = extreme poverty by income 2010, and V15 = Gini index. Bold values represents *p* < 0.05.

## Data Availability

The datasets generated during and/or analyzed during the current study are available from the corresponding author upon request.
